# Malaria Epidemiology in Mersin Province, Turkey from 2002 to 2011

**Published:** 2013

**Authors:** M F Aydin, A Sahin

**Affiliations:** Research Laboratory, Higher Health School, University of Karamanoğlu Mehmetbey, 70200, Karaman, Turkey

**Keywords:** Malaria, Epidemiology, *Plasmodium*, Turkey

## Abstract

**Background:**

Malaria is an infectious disease caused by *Plasmodium* spp. with high morbidity and mortality in human in tropical and subtropical regions. In recent years, number of malaria cases has been significantly reduced because of fight with the disease in Turkey. This study intended to investigate the malaria epidemiology in Mersin Province from 2002 to 2011 using data from the provincial Public Health Directorate.

**Methods:**

Over ten years, 303573 blood samples were taken from the people by active and passive surveillance methods and blood smears were prepared. Smears were stained with Giemsa and examined under the microscope.

**Results:**

Totally, 73 people including 44 male and 29 female were positive in terms of *Plasmodium* spp. It was determined that *P*. *vivax* observed in 67 cases while *P. falciparum* in 6 cases. Cases were mainly observed in 15 to 44 years old range, showed an increase between June-September periods and a significant decrease after 2006. Out of the 73 malaria cases, 54 cases were from Mersin Province and 13 cases were imported from another province of Turkey. Six cases were transmitted from abroad.

**Conclusion:**

These results provide information about malaria epidemiology in an endemic area in Turkey and contribute its prevention in Mersin Province.

## Introduction

Malaria is a public health problem caused by *Plasmodium* species and transmitted by mosquitoes of *Anopheles* genus commonly seen in tropical and subtropical zones globally. People can be infected by infected mosquito bites, by blood transfusion, tissue transplantation and transplacental carry (1). There are more than one hundred *Plasmodium* species infect various mammals. In human, malaria is caused by *P. falciparum*, *P. malariae*, *P. ovale*, *P. vivax* and *P. knowlesi* (2, 3). Among those infectious spp., *P. falciparum* is the most common species throughout the world followed by *P. vivax*. *P. falciparum* is responsible for the majority of deaths while the other species cause a generally milder form of malaria that is rarely fatal (4). Although the most common *Plasmodium* species is *P. vixax*, malaria caused by *P. falciparum* transmitted from abroad have also been observed in recent years in Turkey (5).

In 1957, Turkey participated to malaria eradication program organized by the World Health Organization. Since that date malaria eradication studies have been successful and in 1970 the number of cases has decreased to 1263. Malaria cases have been increased again since 1971. In 1977 and 1994, 115512 and 84345 cases have been observed with large outbreaks respectively (5, 6).

In Turkey some epidemiological studies are conducted about malaria (7–24). The aim of this study was to evaluate malaria cases diagnosed between 2002 and 2011 in the province of Mersin in Turkey.

## Materials and Methods

**Fig. 1:**
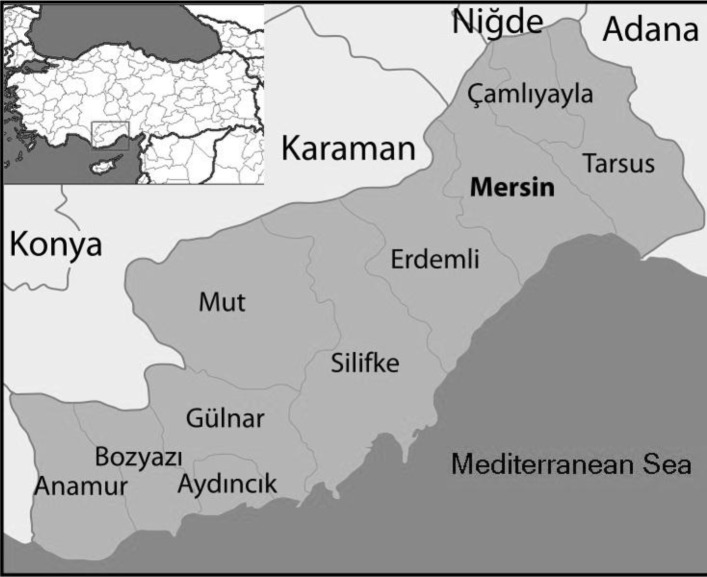
Location map of the province of Mersin in Turkey

The materials of this study were thick drop and thin blood smears prepared from 303573 people who were suspected having malaria by Mersin Public Health Directorate between 2002 and 2011. *Plasmodium* species were investigated under a light microscope magnification X1000 after preparation of blood samples from febrile and afebrile periods. Results were evaluated in terms of years, genders, age groups, seasons, locations and identified species. Locations, where malaria cases were detected in the province of Mersin are presented in Fig. 1.

## Results

Blood samples were taken from 303573 individuals and malaria parasite has been identified in 73 persons (0.02%) including 44 male and 29 female over ten-years. Malaria cases were determined in 0-1 months and 65 years old periods and mostly between 15 to 44 years old people.

**Fig. 2:**
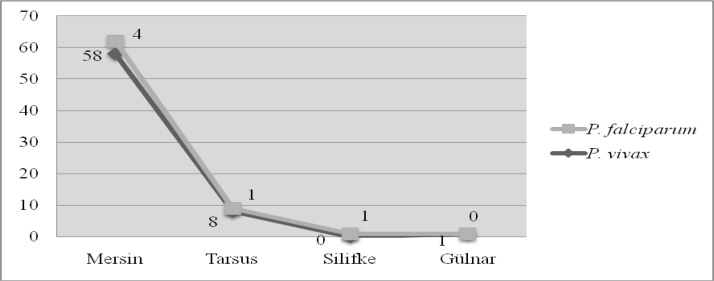
Distribution of *Plasmodium* species according to locations of Mersin Province

**Fig. 3:**
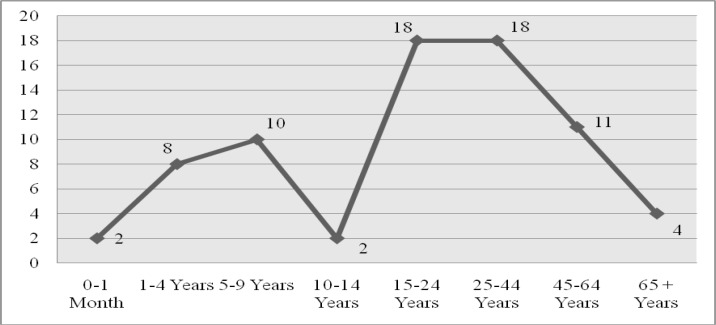
Distribution of malaria cases according to age groups

**Fig. 4:**
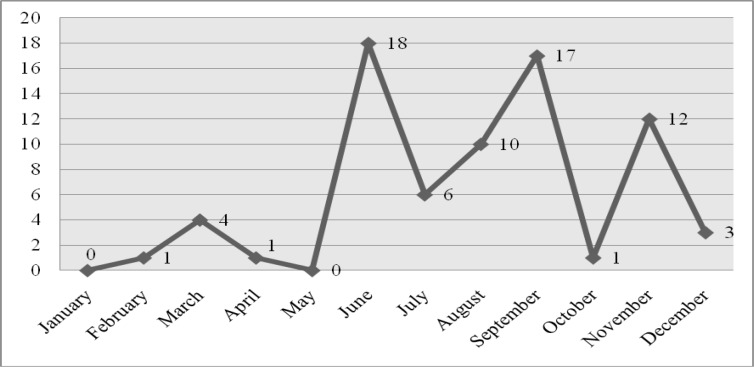
Seasonal distribution of malaria cases in Mersin Province

*P. vivax* and *P. falciparum* species were identified and all cases caused by *P. falciparum* were observed to be transmitted from abroad. Thirteen cases of *P. vivax* malaria transmitted from another province of Turkey. Seasonal distribution of malaria cases has also been determined. The seasonal cases have mostly been found between the years 2002 and 2005 and showed an increase between the months of June-September (Tables 1–2, Figs. 2–4).

**Table 1:** Distribution of malaria cases according to gender and years

Year	Male	Female	Total n (%)
2002	12	4	16 (21.92)
2003	10	6	16 (21.92)
2004	7	9	16 (21.92)
2005	7	7	14 (19.18)
2006	3	-	3 (4.11)
2007	1	3	4 (5.48)
2008	-	-	-
2009	-	-	-
2010	3	-	3 (4.11)
2011	1	-	1 (1.37)
Total	44 (60.27%)	29 (39.73%)	73

**Table 2:** Distribution of malaria cases according to locations

Year	Mersin	Tarsus	Silifke	Gülnar
2002	12	3	1	-
2003	13	3	-	-
2004	15	1	-	-
2005	12	1	-	1
2006	3	-	-	-
2007	3	1	-	-
2008	-	-	-	-
2009	-	-	-	-
2010	3	-	-	-
2011	1	-	-	-
Total	62 (84.93%)	9 (12.33%)	1 (1.37%)	1 (1.37%)

## Discussion

Against the increasing diagnostic and treatment methods and also eradication programs, malaria is still an important infection today globally. While malaria causes moderate infections in people with a strong immune response, it can be fatal for baby, children and pregnant women (25). In Turkey, malaria is still a problem because it creates a burden on the economy and causes loss of work and power (24, 26). However malaria eradication studies started in 1926 caused a decrease in the number of cases (5).

In Turkey two important malaria outbreaks were observed in the years of 1987 and 1994 and after 1996, the incidence of malaria has decreased. In the years of 2002, 2004, 2005, 2006, 2007 and 2008 10224, 5302, 2084, 796, 358 and 215 malaria cases have been determined respectively in Turkey. These results show that malaria cases decreased further every year (6). According to the results of this study, it has been observed that a significant decrease in malaria cases since 2006 in Mersin Province.

Malaria can be seen in both male and female. But it is observed in our study frequently in males (60.27%) and the results of similar studies (7–9) support this data. It is thought that malaria cases were seen more in males due to they are more active than females in working life and exist in the external environment during the evening when mosquitoes are active.

In 91.78% of malaria cases the agent was *P. vivax* and in 8.22% *P. falciparum* was responsible for the diseases. This result is compatible with the data of *P. vivax* is the only endemic species in Turkey (18, 21, 24, 27). Although *P. vivax* is the only endemic species in Turkey, *P. falciparum* is responsible from some cases determined in the country and it is thought that it sourced from abroad (9, 10, 21, 22).

Malaria cases are frequently observed in people between the ages of 15-44 yr and mostly seen in people over the age of 15 yr (15, 16, 21). This shows that the results of other studies conducted on this topic in Turkey are compatible of our study result. It is considered that people over the age of 15 yr travel more than the others for the purpose of business, education and tourism. This situation is a factor increasing the probability of exposure to malaria vector by being outdoors for a long time.

In this study, malaria cases were determined mostly in June (24.65%), September (23.28%), November (16.43) and August (13.69%), respectively. More cases were occurred in the summer months than the other months as a result of ideality for mosquito breeding and the development, having a lot of tourism activity and people being in the outdoor environments (26, 28, 29). Additionally Mersin has subtropical climatic characteristics. So malaria cases were determined in all seasons in Mersin Province of Turkey.

## Conclusion

Malaria cases were viewed throughout the year in Mersin Province because of the climate, the nature, the socio-economic structure and population mobility. It is recommended that malaria screening and vector control must be done throughout the year in Mersin Province and all of Turkey to maintain the decreasing the incidence of malaria.

## References

[1] Cox FE (2010). History of the discovery of the malaria parasites and their vectors. Parasit Vectors..

[2] Alkan MZ, Sönmez TG, Topçu AW, Söyletir G, Doğanay ME (2007). *Plasmodium* species. Infection Diseases and Clinical Microbiology.

[3] Donald JK, Mandell GL, Bennet JE, Dolin R (2000). *Plasmodium* Species (Malaria). Principles and Practice of Infectious Diseases.

[4] Unat EK, Yücel A, Altaş K, Samastı M (1995). Unat's Medical Parasitology.

[5] Özbilgina A, Topluoglu S, Es S, Islek E, Mollahaliloglu S, Erkoc Y (2011). Malaria in Turkey: successful control and strategies for achieving elimination. Acta Trop..

[6] Tugluoglu F (2008). Malaria control in Turkey (1924–1950). Turkiye Parazitol Derg..

[7] Akkafa F, Simsek Z, Dilmec F, Baytak S (2002). Malaria epidemiology in ŞanlIurfa. Turkiye Parazitol Derg..

[8] Altas K, Polat E, Aksın NE, Özcan N, Sevimli AA (1998). Malaria cases determined by Malaria department in Istanbul between 1992-1997. Turkiye Parazitol Derg..

[9] Alver O, Akalın H, Mıstık R, Helvacı S, Tore O (2005). The epidemiology of malaria in Bursa. Turkiye Parazitol Derg..

[10] Alver O, Atıcı E, Tore O (2009). The Investigation of Malaria Cases in Bursa between 2006-2008. Turkiye Parazitol Derg..

[11] Çetinkaya Z, Ozçelik R (2004). Epidemiology of malaria in Afyon. Turkiye Parazitol Derg..

[12] Erensoy A, Kuk S (2010). The epidemiology of malaria in Elazığ and Bingöl between 2005 and 2008. Turkiye Parazitol Derg..

[13] Ertug S, Gurel M, Eyigor M, Doyuran ES (2002). Malaria cases in Aydın region. ADÜ Tıp Fak Derg..

[14] Göz Y, Kurtoğlu MG, Gürsoy M, Aydın A (2004). Malaria in Van: An Epidemiological Study. Türkiye Parazitol Derg..

[15] İnanc T, Kuk S, Yazar S (2012). The Epidemiology of Malaria in Çorum between 2006 and 2011. Kafkas Univ Vet Fak Derg..

[16] Kuk S, Özden M, Kaplan M (2006). The Epidemiology of Malaria in Elazig between 1996 and 2004. Turkiye Parazitol Derg..

[17] Ostan I, Limoncu ME, Tüysüz MA, Köroglu G, Ozbilgin A (2006). Evaluation of malaria cases in Manisa from 2002 to 2004. Turkiye Parazitol Derg..

[18] Sahin IH, Zeyrek FY, Aydın MF, Öntürk H, Basank M (2012). Malaria Epidemiology in Bitlis From 1998 to 2008. Turkiye Parazitol Derg..

[19] Saka G, Ertem M, İlçin E (2000). Malaria in Diyarbakır. Turkiye Parazitol Derg..

[20] Sarı C, Sakarya S, Ertabaklar H, Öncü S, Ertuğ S (2004). Evaluation of Malaria Cases in Aydin between 2001 and 2003. Türkiye Parazitol Derg..

[21] Ser O, Cetin H (2012). Evaluation of Malaria Cases in Antalya between 2001 and 2011. Turkiye Parazitol Derg..

[22] Tamer GS (2008). The Epidemiology of Malaria in Kocaeli. Turkiye Parazitol Derg..

[23] Temiz H, Gül K (2006). Evaluation of Malaria Cases in Diyarbakir between 1999 and 2004. Turkiye Parazitol Derg..

[24] Aslan G, Seyrek A, Kocagoz T, Ulukanligil M, Erguven S, Gunalp A (2007). The diagnosis of malaria and identification of *Plasmodium* species by polymerase chain reaction in Turkey. Parasitol Int..

[25] World Health Organization (2011). World malaria report. http://www.who.int/malaria/world_malaria_report_2011/WMR2011_noprofiles_lowres.pdf.

[26] Alten SB, Çaglar SS, Ozer N (2000). Malaria and its vectors in Turkey. European Mosq Bull..

[27] Saygı G (2002). Fundamental Medical Parasitology.

[28] Ergonul O, Azap A, Akgunduz S (2007). Malaria cases in Turkey: do climatic changes play any role?. Int J Antimicrob Agents..

[29] Canda MS (1991). Ocopathology of Malaria and its importance for our country. Turkiye Parazitol Derg..

